# Comparison of Body, Auricular, and Abdominal Acupuncture Treatments for Insomnia Differentiated as Internal Harassment of Phlegm-Heat Syndrome: An Orthogonal Design

**DOI:** 10.1155/2015/578972

**Published:** 2015-11-10

**Authors:** Yue Jiao, Ying Han, Xin Li, Yi-gong Fang, Zhao-hui Liu, Wen-na Zhou, Jin-cao Zhou, Zhong-chao Wu, Jin-hong Yang, Shao-yuan Li, Fan-ying Meng, Wei-wei Xu

**Affiliations:** ^1^Institution of Acupuncture and Moxibustion, China Academy of Chinese Medical Sciences, Beijing 100700, China; ^2^TCM Hospital of Mentougou District, Beijing 102300, China; ^3^Medical College of Xiamen University, Xiamen 361000, China

## Abstract

*Objective.* To identify the optimum treatment protocol for insomnia among auricular, body, and abdominal needling methods.* Methods.* A three-factor (3 needling protocols) and three-level experimental scheme was designed based on orthogonal method. 54 patients of insomnia differentiated as internal harassment of phlegm-heat syndrome were given two courses of acupuncture treatment (each with 20 times of acupuncture). The therapeutic effects were evaluated by comparing the Pittsburgh sleep quality index (PSQI), Hamilton Depression Scale (HAMD) scores, and Hamilton Anxiety Scale (HAMA) scores of patients before treatment, after one course of treatment, and after two courses of treatment as well as one month after treatment.* Results.* Body, auricular, and abdominal acupuncture treatments all alleviated symptoms of insomnia, depression, and anxiety, but body and auricular acupuncture had stronger therapeutic effects.* Conclusions.* Body acupuncture at basic points shall be given priority in protocol selection for insomnia. The second-best choice is auricular acupuncture with basic points combined with points based on Traditional Chinese Medicine (TCM) theories. Abdominal needling with very quick effect can be an alternative protocol with basic points combined with syndrome differentiation points.

## 1. Introduction

Insomnia is a sleep disorder that includes trouble falling asleep, staying asleep, or waking too early, resulting in daytime impairment [1]. About 30% of adults report at least one of the symptoms of insomnia. When daytime impairment is added as a criterion, the prevalence is about 10% [2]. Long-term insomnia relates to body and mental health impairment, multivisceral dysfunction, and immune decline, as well as depression, anxiety, and other mental diseases [3]. Prevalence is higher among women [4], and, due to their physical and psychological peculiarity, women may easily get depression and anxiety as comorbidities of insomnia.

The sleep-wake cycle is a very complicated process, which involves central and peripheral nervous systems as well as the endocrine system [5, 6]. Modern medicine has not possessed a complete and clear understanding of the pathogenesis so far, whereas it is commonly accepted that pathological changes occur in the anatomical structure of the sleep-wake cycle, including inhibitory nucleus and arousal nucleus and the imbalance of corresponding neurotransmitters. In addition, endocrine regulation is in close relationship with the sleep-wake rhythm, for hormones like melatonin have been confirmed to have a curative effect on insomnia. To put it in a simple way, the pathogenesis manifests either as a weakening of the inhibitory function or as an enhancement of the arousal function.

From a Traditional Chinese Medicine (TCM) perspective, the pathogenesis of insomnia is intricate, involving disharmony of Zang and Fu organs (viscera) and disorder of Wei and Ying Qi (defensive qi and nutrient qi) as well as the Shen (spirit) disturbance of Zang organs. In brief, the pathogenesis of insomnia is regarded as imbalance between Yin and Yang, particularly a result of excessive Yang and deficient Yin, which is quite similar to the general understanding of western medicine.

Acupuncture is a simple but useful treatment for insomnia, with a success rate of around 90% [7]. An explanation of modern medicine is that acupuncture can improve the neurotransmitter balance in the central nervous system. For instance, acupuncture increases the contents of *γ*-amino butyric acid (GABA) and Serotonin in the brain [8–10] and thus improves sleep quality. Improvement may be also due to the endocrine system, for example, the nocturnal increase in endogenous melatonin secretion [11]. Acupuncture treatment has several forms besides body needling, one group of which is micro needling system such as auricular and abdominal needling. Mechanisms underlying auricular and abdominal acupuncture are quite identical to the mechanism of body needling, which is characterized by dredging meridians and collaterals, building up body resistance to pathogenic factors, balancing Yin and Yang, and regulating the functions of Zang and Fu organs.

The study selected patients with phlegm-heat syndrome in TCM diagnosis based on references to traditional syndrome differentiation, clinical experiences, and literature review. As the life rhythm speeds up, more and more people suffer from inadequate sleep and improper diet. Their syndrome manifests as phlegm-heat. In the perspective of TCM, the pathogenesis of insomnia is “deficiency in origin and excess in symptom” (Ben Xu Biao Shi), and in most cases it cooccurs with phlegm and dampness as well as blood stasis [12]. The symptoms of phlegm-heat syndrome are as follows [13]: restless sleep, irritation and upset, suppression in the chest and gastric fullness, bitter taste in the mouth and profuse phlegm, dizziness, red tongue, yellow and thick tongue coating, and rapid pulse or rapid with slippery pulse.

Acupuncture is a complex therapy, including crucial factors such as syndrome differentiation, acupoint combination, and needling method as well as manipulation. Most clinical studies of insomnia treatments use single or combined protocols, whose therapeutic effects vary as different methods and indicators are used. Among Randomized Controlled Trials (RCTs) of acupuncture treatment for insomnia published in recent 10 years, the most commonly reported methods are body needling, auricular needling, abdominal needling, and their combinations. Although different influences are reported due to variances of methods and combination of acupoints, the effective rates in these studies are all highly or equally reported; moreover, there are various methods to form control groups, but the horizontal comparison and superiority trials are scanty. Aiming to identify an optimum protocol for insomnia, based on RCTs in China and abroad in recent ten years, this study chose the 3 highly evaluated protocols with different acupoints formula.

The orthogonal method is the best choice to design an experiment involving multivariate statistics. Its strengths lie in balanced distribution, calculation simplicity, and symmetrical comparability, so that it not only allows for effective experimental results with minimum time and cases of illness in clinical study, but also is capable of showing clearly interaction among factors and superiority among levels of each factor.

This study is designed as an orthogonal test to evaluate the effects of three acupuncture treatments on 54 patients with insomnia differentiated from internal harassment of phlegm-heat syndrome.

## 2. Materials and Methods

### 2.1. Data Collection

54 patients (45 females and 9 males) suffering from primary insomnia diagnosed by Diagnostic and Statistical Manual of Mental Disorders, Fourth Edition [1], and differentiated from phlegm-heat syndrome, all aged between 26 and 60 years (with the average age of 43.9 ± 9.9 years), were enrolled in the study and treated in the Acupuncture Hospital of China Academy of Chinese Medical Sciences from May 2012 to May 2013.

### 2.2. Inclusion and Exclusion Criteria

Inclusion criteria were as follows:

1. Primary insomnia patients diagnosed in accordance with DSM-IV, as mentioned above.
2. Patients with primary insomnia differentiated from phlegm-heat syndrome, diagnosed in accordance with Terminology of Clinical diagnosis and treatment of TCM as mentioned above.
3. Patients aged between 20 and 60 years (including 20- and 60-year olds).
4. The total score of Pittsburgh sleep quality index (PSQI) [14] > 7.
5. The score of Hamilton Depression Scale (HAMD) [15] < 20.
6. The score of Hamilton Anxiety Scale (HAMA) [16] < 14.
7. Patients who signed the informed consent.

Exclusion criteria were as follows:

1. Pregnancy and lactation.
2. Patients with cardiovascular, lung, liver, kidney, or hemopoietic system diseases in late stages.
3. Patients with mental diseases.
4. Patients accepting related medication or other treatments.
5. Severe adverse reactions to acupuncture (e.g., fainting during acupuncture).

### 2.3. Method

#### 2.3.1. Grouping

The L_9_ (3^4^) orthogonal array (Table 1) was used in the study. Patients were randomly assigned to nine protocols in a single-blinded study. In Table 1, alphabets A, B, and C denote different treatment protocols and values 1, 2, and 3 indicate three different levels; alphabet D denotes the error term, which is excluded in the study. The details of protocols and levels employed in this paper are shown in Table 2.

#### 2.3.2. Treatment

Eight Chinese-trained and licensed acupuncturists with a median of 12 years of experience (range: 5 to 20 years) provided study treatments in the clinics. Before the study, all acupuncturists made consensus on the protocol of point selection and needle manipulations.

*(1) Body Acupuncture*. The acupuncture point selections were based on Traditional Chinese Medicine meridian theory to treat insomnia. The points were made references to the* evidence-based guidelines of clinical practice with acupuncture and moxibustion: insomnia* [17] and approved by committee of acupuncturists of the study.

*Acupoints*. The basic acupoints are Zhaohai (KI 6), Shenmai (BL 62), Shenmen (HT 7), Yintang (EX-HN 3), Sishencong (EX-HN 1), and Anmian (Ex-HN 22).

Acupoints selected on the basis of syndrome differentiation (hereafter referred to as syndrome differentiation acupoints) are Fenglong (ST 40), Neiting (ST 44), and Quchi (LI 11).

All acupoints were selected bilaterally except Yintang (EX-HN 3).

*Operation*. 1 cun (0.25 × 25 mm) and 1.5 cun (0.25 × 40 mm) disposable needles were used in the treatment. Patients laid in the supine position as the treatment proceeded. Needles were inserted with lifting and thrusting as well as rotating methods till qi sensation was felt. Among acupoints, Zhaohai (KI 6) was stimulated by the reinforcing technique, while Shenmai (BL 62) and syndrome differentiation acupoints were stimulated by the reducing technique. The rest of the acupoints were stimulated by the neutral reinforcing and reducing technique.

All points were stimulated for 1 minute in every 15 minutes. Needles were retained for 30 minutes.

*Frequency and Courses of Treatment*. Frequency and courses of treatment were as follows: once every other day, 3 times per week, 10 times in each course, and 2 consecutive courses in total.

*(2) Auricular Acupuncture*. The auricular point selections were based on TCM theory. The points were also made references to* evidence-based guidelines of clinical practice with acupuncture and moxibustion: insomnia* [17] and were approved by committee of acupuncturists of the study.

*Acupoints*. We used auricular acupoints as suggested in the* nomenclature and location of auricular points* [18].

The basic acupoints are Shenmen (TF4), Occiput (AT3), Chuiqian (LO4), and Subcortex (AT4).

Acupoints based on TCM theory were Spleen (CO13), Stomach (CO4), Heart (CO15), and Liver (CO12).

Acupoints were selected unilaterally at a time, and alternated every other day.

*Operation*. 1 cun (0.25 × 25 mm) disposable needles were used in the treatment. Patients took the supine position. After routine disinfection of acupoints, the needle was inserted and manipulated moderately without penetrating the skin.

The frequency and courses of treatment were the same as those of the body acupuncture.

*(3) Abdominal Acupuncture*. The abdominal acupuncture was guided by TCM theory and as one of holographic acupuncture methods. We used abdominal acupoints as suggested in the* Abdominal Acupuncture* [19].

*Acupoints*. Basic formula is as follows: Zhongwan (RN 12), Xiawan (RN10), Qihai (RN 6), and Guanyuan (RN 4); Shangqu (KI 17) (bilateral), Huaroumen (ST 24) (bilateral), Wailing (ST26) (bilateral), and Pinggan (extra point) (Bilateral).

Syndrome differentiation acupoints were Daheng (SP 15) (bilateral), Xiere (extra point) (bilateral).

Location of Pinggan was 0.7 cun outside and 0.3 cun above Huaroumen (ST 24); Xiere was 3 cun outside of Qihai (RN 6).

*Operation*. 1 cun (0.22 × 25 mm) and 1.5 cun (0.25 × 40 mm) disposable needles were used in the treatment. Patients took the supine position. After routine disinfection of acupoints, needles were vertically inserted into the skin according to the following sequence: from upward to downward, from inside to outside.

Needles were applied at Zhongwan (RN 12), Xiawan (RN10), Qihai (RN 6), and Guanyuan (RN 4), touching the linea alba of Ren meridian (fascia layer) and after needles were inserted at Zhongwan (RN 12), Xiawan (RN10), and Qihai (RN 6), the direction of needle tips was adjusted to be inserted obliquely at an angle of 45° along the Ren Meridian, while a needle was inserted vertically at Guanyuan (RN 4).

After a needle was applied at Shangqu (KI 17), the direction of the needle tip was adjusted to move obliquely downward at an angle of 45° along the kidney meridian to the depth of the fat layer.

Needles were inserted vertically at Huaroumen (ST 24), Wailing (ST26), Pinggan, Daheng (SP 15), and Xiere, to the fat layer at Huaroumen (ST 24), Wailing (ST 26), and Daheng (SP 15), to the muscle layer at Pinggan and Xier.

The frequency and courses of treatment were the same as those of the body acupuncture.

Patients were kept warm during treatment.

### 2.4. Clinical Outcomes

Sleep quality of patients was evaluated with PSQI scores before the treatment and after 1 course and 2 courses of treatment and 1 month after treatment. Similarly, depression was evaluated with HAMD scores and anxiety with HAMA scores.

### 2.5. Statistical Analysis

SPSS17.0 was used to analyze data. A descriptive analysis was performed first, and then was followed by a visual calculation in accordance with requests of orthogonal test. The *R* value indicated the influences of different protocols; that is to say, a higher score of *R* meant the protocol was more effective.

## 3. Results

### 3.1. General Data

There was no statistically significant difference between the groups in terms of age, duration of disease, and scores of PSQI, HAMD, and HAMA. The average age, duration of disease, and PSQI, HAMD, and HAMA scores were 41.96 ± 11.80 (years), 4.26 ± 5.3 (years), and 12.59 ± 3.21, 10.83 ± 4.32, and 8.44 ± 3.92, respectively (Table 3).

### 3.2. PSQI Scoring

**Figure 1:**
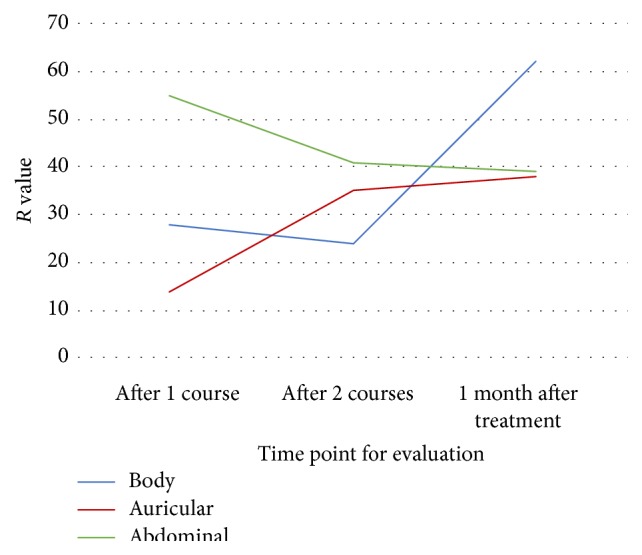
Influences on PSQI scores of different protocols at each time point.

The *R* values symbolizing the range of PSQI score in orthogonal test were shown in Figure 1. As time went by, auricular acupuncture demonstrated an accelerating trend on PSQI score reduction, while abdominal acupuncture showed a decelerating trend. Body acupuncture presented an impressive promotion after treatment rather than during the treatment (Figure 1).

Table 4 compares the PSQI score reduction from three levels of three protocols. It indicates that A2 basic acupoints, B3 basic points + acupoints based on TCM theories, and C2 basic + syndrome differentiation acupoints had stronger influences on the descending trend of PSQI scores, which meant continuous improvement of sleep quality (Table 4).

### 3.3. HAMD Scoring

**Figure 2:**
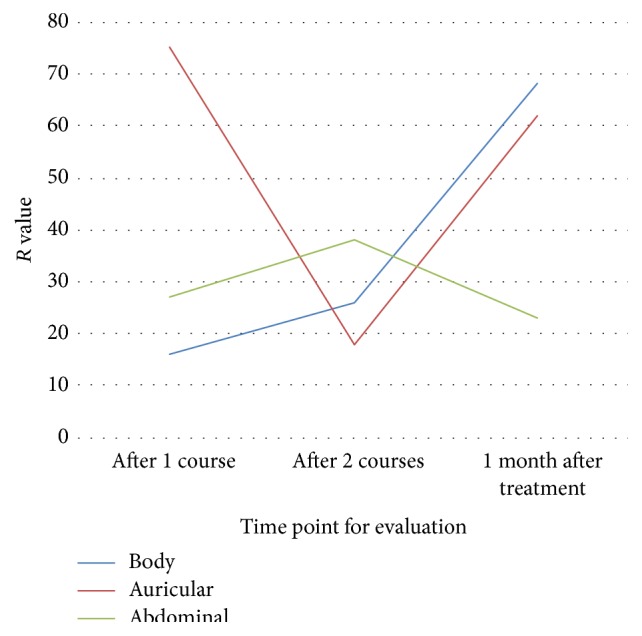
Influences on HAMD scores of different protocols at each time point.

The *R* values symbolizing the range of HAMD score in orthogonal test were shown in Figure 2. As time went by, auricular acupuncture demonstrated first a sharply decelerating and then accelerating trend on HAMD score reduction, while abdominal acupuncture showed an accelerating and then decelerating trend. Body acupuncture presented an impressive promotion especially after the treatment.

Table 5 compares the HAMD score reduction from three levels of three protocols. It indicates that A2 basic acupoints, B3 basic points + acupoints based on TCM theories, and C2 basic + syndrome differentiation acupoints had stronger influences on the descending trend of HAMD score, which meant improvement in depression (Table 5).

### 3.4. HAMA Scoring

**Figure 3:**
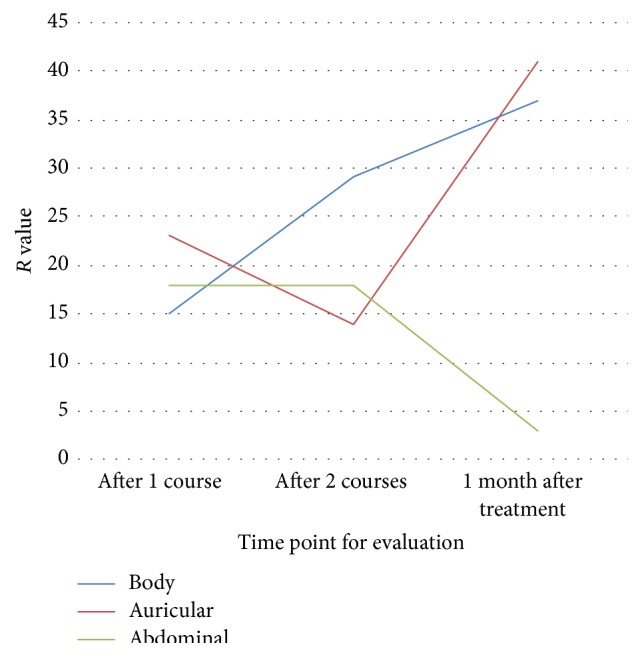
Influence on HAMA scores of different protocols at each time point.

The *R* values symbolizing the range of HAMA score in orthogonal test were shown in Figure 3. As time went by, auricular acupuncture demonstrated a slightly decelerating and then accelerating trend on HAMD score reduction, while abdominal acupuncture showed a decelerating trend, whereas body acupuncture presented an accelerating trend.

Table 6 compares the HAMD score reduction from three levels of three protocols. It indicates that A2 basic acupoints, B1 basic acupoints, and C2 basic + syndrome differentiation acupoints had stronger influences on the descending trend of HAMA score, which meant improvement in anxiety (Table 5).

## 4. Discussion

The study used orthogonal design, an experimental design used to test the comparative effectiveness of 3 intervention components (protocols), each of which took on 3 variants (levels) in this study, because it allowed testing the effectiveness of body, auricular, and abdominal acupuncture simultaneously in a single study with far fewer experimental units than it would take to exhaust all possible intervention combinations [20]. To design and implement this technique, orthogonal array would first be chosen to fit the study purpose. Since 3 kinds of acupuncture were tested for interaction, and point selection was the second consideration, then it would be structured to the 3 factors (the number of columns in an array) and 3 levels (the maximum number of values that can be taken on by any single factor) of the array. With respect to suitable orthogonal array selection (orthogonal arrays are most often named following the pattern L_Runs_ (Levels^Factors^)), the rule is to find one with the smallest number of runs (the number of test cases) [21]. Since there was no orthogonal array of 3 factors and 3 levels, then L_9_ (3^4^) orthogonal array was most suitable in the study.

Among the three protocols, body needling had steadily accelerating effectiveness on improvement of insomnia, depression, and anxiety in both the treatment and the follow-up period. Therefore it was the most recommended therapy and the best level was to use basic acupoints for treatment. The mechanism of body needling treatment for insomnia in TCM included regulating Yin Qiao and Yang Qiao meridians with points of Zhaohai (KI 6) and Shenmai (BL 62) to balance Yin and Yang based on the Yin-yang theory and Ying-wei theory; tonifying the heart and calming the mind with Shenmen (HT 7), Yintang (EX-HN 3), Sishencong (EX-HN 1), and Anmian (Ex-HN 22) based on the shen- (spirit-) dominating-sleep theory. The basic points alone were able to adjust the function of the whole body, so that was enough to take effect on insomnia patients with internal harassment of phlegm-heat syndrome. However, further studies are needed to explore suitable protocols for insomnia patients with other syndromes.

In TCM, heart is the master of five viscera including heart, lung, spleen, liver, and kidney. “Shen of heart,” dominating mental activity, is self-consciousness and unique human ability. Their function however quite resembles cerebrum. If shen calms down, humans fall asleep, while when shen activates, they wake up. Moreover, sleep follows daily biological rhythm and shows as a sign of balance of Yin and Yang. In TCM, if wei qi flourishes in Yang qiao meridian, eyes open and consciousness is regained, while if wei qi flourished in Yin qiao meridian, eyes close and sleep will come. According to meridian circulation, Yin qiao and Yang qiao meridians confluence at inner canthus and then enter the brain. In other words, they are both crucial routes related to sleep. Thus it can be seen that TCM theory considers the fact that sleep is closely linked with brain function, so that most of acupoints selected to treat insomnia locate on the head. In TCM, the insomnia treating principle lies in balancing Yin and Yang. And fMRI can be taken as one of parameters of insomnia regulations by acupuncture. Gao et al. [22] reported that, in sleep deprivation, an imbalance occurs; acupuncture stood for a homeostatic force to renormalize the Yin and Yang; biphasic regulation effects of acupuncture, the salience network, composed of the anterior insular cortex and anterior cingulate (ACC), a unique interoceptive autonomic circuit, might indicate the mechanism underlying acupuncture in restoring sleep deprivation. Effects of acupuncture were closing to launch homeostatic regulation [23, 24].

The auricular needling also had a clear therapeutic effect on insomnia, depression, and anxiety. Unlike body needling, its efficacy in treating insomnia increased continuously, while, in ameliorating depression and anxiety, the increase got decelerated in the treatment period while it was accelerated during follow-up period. From the respective of TCM, the auricle had close relationships with both meridians and viscera. Holographic medicine and modern medicine have also confirmed the somatotopic function of specific regions of the ear. Various nerves including the spinal and cranial nerves are distributed at the auricles, particularly in the triangular fossa and the concha region. Among those nerves, the vagus nerve is a mixed nerve composed of about 80% afferent fibers. The concha area has a rich distribution of the vagus nerve. Stimulating points in this region with needles can adjust functions of the viscera and the central nervous system, as well as the autonomous system [25–27]. It was speculated that the insomnia regulating effects of vagus nerve stimulation were partially attributed to the projection of afferent fibers to the nucleus tractus solitarius, which was further connected directly and indirectly with brain structures [28] including the locus coeruleus, cerebral cortex, hippocampus, thalamus, and cerebellum [29]. Just yet, some of these brain regions were also believed to be involved in the pathogenesis of sleep disorder [30–32], which built a basis for treatment with vagus nerve stimulation [29]. Moreover, excitation of the parasympathetic nerve, which was part of the vagus nerve, stimulated the pineal gland to secrete melatonin [33–37]. Insomnia closely related to functional decrease of central melatonin; mutual promotion between excitation of parasympathetic nerve and melatonin secretion became a basis of treating insomnia [38–41]. It might partly explain the therapeutic effect of auricular acupuncture. Furthermore, basic points like Occiput (AT3), Chuiqian (LO4), and Subcortex (AT4) were mainly used as corresponding points of the brain. Stimulation at these points and Shenmen (TF4) calms the mind. Points based on TCM theory such as spleen (CO13), stomach (CO4), heart (CO15), and liver (CO12) can regulate viscera functions to improve phlegm-heat symptoms, and since they are all located in the concha region, these points can improve sleep quality by stimulating the vagus nerve. Besides, the stimulation of auricular needling was stronger than the other two protocols, so it may take effect very quickly, especially for the treatment of anxiety and irritation, and it might explain why it had rapid and solid effect in depression and anxiety.

Brain imaging tools have been used to investigate the fMRI signal change evoked by transcutaneous vagus nerve stimulation recently [42]. Kraus et al. [42] found that robust tVNS can induce fMRI signal decreases in limbic brain areas, including the amygdala, hippocampus, parahippocampal gyrus, and middle and superior temporal gyrus, as well as an fMRI signal increase in the insula, precentral gyrus, and thalamus. As mentioned above, these were all important structures related to sleep-wake cycle. In recent study of tVNS treating major depressive disorder, by the help of fMRI, Fang et al. [43] found that significant functional connectivity (FC) between default mode network (DMN) and brain regions such as the parahippocampus, ACC, and medial temporal areas. These results suggested that the modulation of tVNS was not targeted at one particular region but it rather influenced brain region networks associated with emotion/affect regulation. Since insomnia also related intimately to depression and anxiety, it might provide a reference approach for treatment and a mechanism explanation.

Abdominal needling could improve insomnia, depression, and anxiety with a gradually decelerated increase. The abdominal region is where the viscera and meridians are located, including the Ren, kidney, spleen, and stomach as well as gallbladder and branch meridian of Du meridians. The abdominal needling helped to harmonize Yin and Yang and regulate meridians and viscera, which corresponded to the pathogenesis of insomnia: imbalance of Yin-Yang and disorder of viscera function. Furthermore, the abdominal needling demanded slight and shallow stimulation, with finer needles, which minimized pain compared with traditional needling techniques, so it was easily accepted by patients, particularly at the initial stage of the treatment. That might explain its remarkable effect at the beginning of treatment. Basic formula combined with syndrome differentiation points produced a stable and better effect. Among the points, Daheng (SP 14) and Xiere are located on and near the spleen meridian, which might strengthen the spleen's function of dispersing phlegm. Wang [44] applied abdominal needling treatment for depression; the author observed that, by means of fMRI-ReHo, patients suffered abnormal changes in several brain regions in resting-state, especially limbic-cortical-striatal-pallidal-thalamic (LCSPT) circuit; however, after abdominal needling treatment, their function could be regulated through amelioration of blood oxygen and metabolism. The circuit and cortico-striato-thalamic-cortical loop (CSTC loop) overlapped quit a lot in the brain. The latter was known to regulate cortical arousal by controlling the effectiveness of the thalamic filter; this system regulated cortical arousal by filtering out sensory input for maintaining sleep or by allowing specific sensory input to the cortex for maintaining cortical arousal [30]. Therefore, one explanation for the fact that abdominal needling took effect on insomnia might lay in the circuit mentioned above.

It shall be noted that our study had several limitations. Firstly, the syndrome differentiation of patients in the study was internal harassment of phlegm-heat syndrome, so the conclusions shall not be applied to other syndromes. Secondly, though the study efficiently compared the therapeutic effects of three protocols and corresponding points selection with a L_9_ (3^4^) table of orthogonal design, it did not examine the interaction effects between them. Therefore, the conclusions did not address the question of whether the protocols shall be used together or separately. Thirdly, both the auricular and abdominal needling showed a significant therapeutic effect at the beginning of treatment, but the increasing effect of auricular needling was unsteady for depression and anxiety and so was the increasing effect of abdominal needling. Differences between body needling and micro-needling system, as well as between different micro-needling systems, need further studies. And finally, the sample in this study was relatively small. The mechanism needs to be researched with more diversified patients and sophisticated experimental designs from both the TCM and modern medicine perspectives.

## 5. Conclusions

The following conclusions can be drawn from this study:

1. Body, auricular, and abdominal needling all improved insomnia and depression as well as anxiety for patients with internal harassment of phlegm-heat syndrome, with the first two protocols being more effective.
2. Among the three protocols, body needling is firstly recommended for it consistently improved insomnia and depression as well as anxiety, and the therapeutic effect kept improving as time went by. The recommended points are basic ones.
3. Auricular needling improved insomnia with a sustainably growing trend, but, for depression and anxiety, within the treatment period, the increasing is decelerated than that during follow-up period. The recommended points are basic points combined with points based on TCM theory.
4. Abdominal needling improves insomnia with a decelerated increase, and, for depression and anxiety, the increase is first accelerated within treatment period, then decelerated during follow-up period. The recommended points are basic ones combined with syndrome differentiation points.

## Figures and Tables

**Table 1:** L_9_ (3^4^) orthogonal array.

Patient number	Column number			
	A	B	C	D
1	1	1	1	1
2	1	2	2	2
3	1	3	3	3
4	2	1	2	3
5	2	2	3	1
6	2	3	1	2
7	3	1	3	2
8	3	2	1	3
9	3	3	2	1

**Table 2:** Designs for protocols and levels.

Protocols	Levels
A: body acupuncture	Level 1: 0 (body acupuncture not applied)
	Level 2: basic acupoints
	Level 3: basic points + syndrome differentiation acupoints

B: auricular acupuncture	Level 1: basic acupoints
	Level 2: 0 (auricular acupuncture not applied)
	Level 3: basic points + acupoints based on TCM theories

C: abdominal acupuncture	Level 1: basic acupoints
	Level 2: basic points + syndrome differentiation acupoints
	Level 3: 0 (abdominal acupuncture not applied)

**Table 3:** Baseline data of patients with insomnia.

Group	Cases	M	F	Age (years)	Duration of disease (years)	PSQI	HAMD	HAMA
1	6	1	5	43.83 ± 11.05	3.42 ± 3.58	13.67 ± 3.01	10.67 ± 4.03	7.83 ± 4.17
2	6	1	5	44.17 ± 10.67	2.33 ± 3.12	11.67 ± 3.93	10.50 ± 2.43	7.83 ± 3.13
3	6	1	5	40.00 ± 15.87	1.58 ± 2.10	14.83 ± 1.60	12.00 ± 5.10	8.33 ± 3.50
4	6	1	5	44.00 ± 14.25	5.21 ± 7.46	13.33 ± 4.18	11.50 ± 6.63	8.50 ± 3.83
5	6	1	5	41.67 ± 7.76	4.25 ± 5.49	12.33 ± 3.14	13.50 ± 4.64	10.50 ± 5.21
6	6	1	5	43.83 ± 13.00	7.75 ± 7.07	12.00 ± 3.29	9.17 ± 2.32	8.33 ± 3.27
7	6	1	5	37.17 ± 10.98	2.58 ± 3.77	9.83 ± 2.86	10.67 ± 3.56	9.83 ± 3.25
8	6	1	5	41.50 ± 12.18	2.50 ± 2.56	11.50 ± 3.45	8.00 ± 4.90	6.67 ± 5.24
9	6	1	5	41.50 ± 10.41	1.19 ± 1.58	14.17 ± 3.43	11.50 ± 5.24	8.17 ± 3.66
Sum	54	9	45	41.96 ± 11.80	4.26 ± 5.34	12.59 ± 3.21	10.83 ± 4.32	8.44 ± 3.92

**Table 4:** PSQI comparison among three levels of each protocol (factor).

Group	After 1 course	After 2 courses	1 month after treatment
A1	79	116	111
A2	87	104	134
A3	75	115	81
B1	75	116	101
B2	81	104	92
B3	85	115	133
C1	52	98	91
C2	109	132	125
C3	80	105	110

**Table 5:** HAMD comparison among three levels of each protocol (factor).

Group	After 1 course	After 2 courses	1 month after treatment
A1	57	90	85
A2	66	93	109
A3	50	67	41
B1	40	82	57
B2	29	75	59
B3	104	93	119
C1	45	92	66
C2	72	98	88
C3	56	60	81

**Table 6:** HAMA comparison among three levels of each protocol (factor).

Group	After 1 course	After 2 courses	1 month after treatment
A1	33	76	78
A2	48	72	95
A3	46	47	58
B1	36	73	94
B2	34	59	53
B3	57	63	84
C1	47	73	76
C2	49	67	79
C3	31	55	76
